# Single-center evaluation of a next generation fully repositionable and retrievable transcatheter aortic valve replacement

**DOI:** 10.1186/s12872-019-1021-7

**Published:** 2019-02-26

**Authors:** Karolina Berntorp, Sasha Koul, Shahab Nozohoor, Jan Harnek, Henrik Bjursten, Matthias Götberg

**Affiliations:** Department of Cardiology, Clinical Sciences, Lund University, Skane University Hospital, 221-85 Lund, SE Sweden

**Keywords:** Transcatheter aortic valve replacement, Paravalvular leak, Permanent pacemaker

## Abstract

**Background:**

The mechanically expandable Lotus Valve System is a fully repositionable and retrievable valve with an adaptive seal to minimize paravalvular leak (PVL). The aim of this study was to evaluate the short- and long-term safety and efficacy of the new device with focus on a new implantation technique to reduce the need for a permanent pacemaker (PPM) post procedure.

**Methods:**

We performed a prospective single-center, non-randomized evaluation of the Lotus Valve System. The first 100 consecutive Lotus Valve implantations were included in the analysis. Outcome was assessed according to VARC2-criteria. Postoperative pacemaker rates were assessed using the national pacemaker registry and electronic medical records. Mortality at 30 days and 12 months were acquired from the national population registry.

**Results:**

Mean age was 82.7 ± 5.6 years, mean Euroscore I was 25.3 ± 14.5%, mean STS-score was 6.5 ± 4.1% and mean aortic valve area was 0.6 ± 0.1 cm^2^. There were no cases of valve embolization, ectopic valve deployment or additional valve implantation. Device success according to the VARC2-criteria was 97%. The 30-day mortality rate was 3%. Two deaths occurred due to stroke and one due to a ventricular rupture. Major stroke rate was 2% and major vascular complication rate was 2%. The 12-month mortality rate was 14%. At discharge 87% of patients had no/trace PVL, 12% had mild PVL and one patient had a moderate PVL. A total of 13% received a new PPM post valve implantation. Among patients who did not have a PPM before the procedure, the PPM rate was 15.3%.

**Conclusions:**

This single-center evaluation of the Lotus Valve System demonstrated a good clinical outcome with a low mortality, in a high-risk population. Introduction of a new implantation technique resulted in lower PPM rates than previously reported without negatively affecting PVL.

**Trial registration:**

Current Controlled Trials ISRCTN14952278, retrospectively registered 06/11/2017.

## Background

Transcatheter Aortic Valve Implantation (TAVI) has become a recommended alternative for severe symptomatic aortic stenosis in patients deemed to be in intermediate or high risk for surgical aortic valve replacement (SAVR), and is standard therapy when surgery is denied. Compared with SAVR, TAVI provides similar or improved outcome in large randomized clinical studies involving primarily patients deemed at intermediate or high risk for SAVR [1–6].

The new mechanically expandable Lotus Valve System enables a precise positioning due to the ability to reposition or fully retrieve the valve even in a locked final state before detaching the bioprosthetic valve from the delivery catheter. The Lotus Valve System also has an adaptive seal to potentially reduce paravalvular leak (PVL) [7, 8]. Reducing PVL is an important feature of modern valves since residual moderate or severe PVL after valve implantation has been demonstrated to be an important negative predictor of long-term outcome [9, 10].

A well-known adverse event to TAVI is the need of a permanent pacemaker (PPM) after implantation due to mechanical interaction of the valve on the electrical conduction system, involving the atrioventricular (AV) node and/or left bundle, causing 2nd degree or 3rd degree AV block [1, 8, 11]. Available evidence on predictors of a PPM implantation are derived from small studies but 1st degree AV block, left anterior hemiblock (LAH) and right bundle branch block (RBBB) have been suggested [12, 13]. The Lotus Valve System has been associated with a relatively high incidence of PPM ranging from 24 to 36% in different studies [7, 8, 11, 14]. After gaining initial experience, we introduced a new implantation technique to address the high PPM rate. The technique was based on avoiding valve oversizing relative to the annulus, potentially minimizing the interaction between the valve and the left ventricular outflow tract (LVOT). Furthermore, we actively sought a high implantation of the valve in a more aortic position, minimizing interaction with the electrical conduction system.

We evaluated the procedural, short- and long-term safety and efficacy of the Lotus Valve System in an all-comers population with severe symptomatic aortic stenosis at our center, according to the Valve Academic Research Consortium – 2 (VARC-2) criteria [15].

## Methods

### Study design

We prospectively collected information with the intent to evaluate the safety and efficacy of the mechanically expandable Lotus Valve System (Boston Scientific, Marlborough, MA, USA) in the first 100 patients with a Lotus Valve implanted at Skåne University Hospital, Lund, Sweden. The Lotus Valve System was used in an all-comers patient cohort, after heart team acceptance when deemed anatomically feasible. Limiting factors for use of the Lotus Valve was an annulus > 27 mm and inadequate vascular access precluding transfemoral approach. The primary outcome measure was device success according to the VARC-2 criterion defined as the absence of procedural mortality and correct positioning of a valve. The secondary outcomes were all-cause mortality, major stroke, major vascular bleeding and a new PPM before discharge, according to the European Society of Cardiology (ESC) guidelines [16]. The study was retrospectively registered at current Controlled Trials ISRCTN14952278, 06/11/2017.

### Patient selection, procedure and follow-up

All patients were assessed to have severe symptomatic aortic stenosis with an indication for TAVI by two multidisciplinary heart team meetings consisting of at least one cardiologist and a cardiac surgeon. Patient risk for surgical valve replacement was based on the Society of Thoracic Surgeons (STS) score for mortality and Euroscore I. All patients went through a pre-procedural angiography and electrocardiogram (ECG)-gated computed tomography for valve sizing and assessment of vascular access. Three different independent experienced cardiologists performed transthoracic echocardiogram (TTE) measurements. PVL and measurements of mean aortic gradient and left ventricular ejection fraction (LVEF) were performed on all patients before discharge. PVL was graded into no/trace, mild, moderate and severe as described elsewhere [15]. Follow-up was conducted according to local clinical practice at the referring hospital. TTE was not performed by a central core lab.

The proctored implantation technique of the Lotus Valve System involves early flaring of the lower portion of the valve in a deep position in the LVOT followed by pulling on the catheter, seating the Lotus Valve into the final position as it expands. After gaining initial experience, we utilized a modified technique which involved limited flaring of the Lotus Valve before seating the valve in a more aortic position with the aim of a final implantation depth of 0–6 mm, or in other words never to allow the frame to come deeper than 0–6 mm into the LVOT. However, this was done without allowing the cranial part of the Lotus Valve frame to cover the coronary ostia. With this technique the bottom of the Lotus Valve seldom reached 10 mm below the annulus during implant.

### Data sources and statistical analysis

This study conforms to the CONSORT guidelines. The pre- and post-operative data were retrieved from electronic medical records. All-cause mortality rate was assessed using the national population registry, which has a 100% follow-up of all citizens. PPM rates were assessed using the national pacemaker registry and electronic medical records. Continuous variables are presented as mean ± standard deviation (SD). Categorical variables are presented as counts and percentages. Comparisons were made using t-test for continuous variables and the chi-square test for categorical variables. A *p*-value < 0.05 was considered statistically significant. For statistical analysis the IBM SPSS Statistics 23 was used.

## Results

### Patients

Between September 2013 to November 2015, 100 patients with an implanted Lotus Valve System were included. Baseline, procedural and follow-up data at 30-days were available for all patients (Tables 1, 2, 3). The mean age was 82.7 ± 5.6 years with 44% being male. The mean Euroscore I was 25.3 ± 14.5%, mean STS-score was 6.5 ± 4.1%. The mean aortic valve area at baseline was 0.6 ± 0.1 cm^2^ (Table 4). The majority of patients (79%) were in New York Heart Association (NYHA) class III or IV.

**Table 1:** Patient characteristics at baseline (*N* = 100)

Age - yrs. mean (± SD)	82.7 (5.6)
Female sex - n (%)	56 (56)
Medically treated diabetes - n (%)	30 (30)
Arterial hypertension – n (%)	91 (91)
Atrial fibrillation - n (%)	42 (42)
Previous Percutaneous Coronary Intervention - n (%)	23 (23)
Previous Coronary Artery Bypass Grafting - n (%)	22 (22)
Previous stroke - n (%)	14 (14)
Chronic renal failure - n (%)	33 (33)
Pulmonary hypertension (severe) - n (%)	28 (28)
Porcelain aorta – n (%)	16 (16)
New York Heart Association functional class III or IV – n (%)	79 (79)
Society of Thoracic Surgeons score - % mean (± SD)	6.5 (4.1)
Euroscore - % mean (±SD)	25.3 (14.5)
Permanent pacemaker at baseline - n (%)	15 (15)

**Table 2:** Procedural data (N = 100)

	Percent
Transfemoral approach	95
Transaortic approach	5
Bicuspid aortic stenosis	3
Valve in valve procedure	1
Severe aortic regurgitation	1
General anesthesia	46
Predilatation	48
Postdilatation	1
Prothesis size distribution 23/25/27 mm	32/29/39

**Table 3:** Procedural and clinical outcome

	n/N (%)
Device success	99/102 (97)^a^
Successful valve retrieval if attempted	2/2 (100)
Coronary obstruction	0/100 (0)
Valve migration	0/100 (0)
Valve embolization	0/100 (0)
Ectopic valve deployment	0/100 (0)
TAVI-in-TAV deployment	0/100 (0)
Procedural mortality	1/100 (1)
30 day mortality	3/100 (3)
Major stroke	2/100 (2)
Major vascular complications	2/100 (2)

^a^Absence of procedural mortality, successful access, delivery, deployment and system retrieval

**Table 4:** Echocardiogram characteristics at baseline, discharge and 1 year

	Baseline (± SD)	Discharge (± SD)	1 year (± SD)^a^	*p*-value 1 year compared to discharge
Mean LVEF %	44.05 (11.1)	48.9 (9.6)	50.5 (7.9)	0.17
Mean aortic gradient mmHg	47.2 (0.1)	9.9 (3.4)	12.2 (4.6)	0.08
Mean aortic valve area cm^2^	0.6 (0.1)	–	–	
	Baseline	Discharge	1 year^a^	*p*-value 1 year compared to discharge
PVL				0.06
None/Trace %	–	87	90.2	
Mild %	–	12	5.9	
Moderate %	–	1	0	
Severe %	–	0	2	

^a^One year follow-up data were available for 52% of the patients

### Device failure and secondary outcomes

Transfemoral approach was used in 95% of the implants and 5% had a transaortic approach. Conscious sedation was used in 54% of patients (Table 2). One patient planned for a Lotus Valve System was converted to an Edwards Sapien 3 valve due to severe aortic tortuosity with inability to track the delivery catheter. In one patient a device failure occurred when a reposition was performed. The device was successfully retrieved but with a femoral access complication. The patient received a new Lotus Valve System 42 days later (Table 3). There were two cases of major vascular complication, requiring surgical intervention. There were no cases of coronary obstruction, valve embolization, ectopic valve deployment or additional valve implantation (Table 3). The procedural mortality rate was 1% due to a ventricular rupture in a patient who had been treated with steroids for many years. In addition, two patients suffered from periprocedural fatal ischemic strokes, probably from calcium dislodgement, resulting in an overall 30-day mortality rate of 3%. All-cause mortality at one year was 14%, representing one new cardiac death, 7 new non-cardiac deaths and three deaths of unknown cause.

### Echocardiographic follow-up

The TTE examination at discharge was performed in 100% of the patients and LVEF, mean aortic gradient and PVL were measured. One year follow-up TTE examination measurements and follow-up were not available for all patients due to differences in follow-up routines between referring hospitals. After one year TTE measurements were available in 52% of the patients. The mean aortic gradient was 47.2 ± 0.1 mmHg at baseline, 9.9 ± 3.4 mmHg at discharge and 12.2 ± 4.6 mmHg at one year, *p* = 0.08 (Table 4). The mean LVEF was 44.1 ± 11.1% at baseline, 48.9 ± 9.6% at discharge and 50.5 ± 7.9% at one year, *p* = 0.17.

The rate of PVL after TAVI was no/trace in 87% and mild in 12% of the patients at discharge (Table 4). One patient had a moderate PVL due to an annular calcified nodule (9 × 7 mm). Similar rates of PVL were observed at one year with no/trace in 94.1% and mild in 3.9% of patients. One patient presented 5 months post TAVI with a severe PVL due to an aortic root abscess. The patient was successfully treated by antibiotics with good outcome.

### Pacemaker and conduction disturbances

Before implantation 15% of the patients had a preexisting pacemaker. After implantation, but before discharge, 13 patients (13%) received a new PPM according to ESC guidelines. Among patients who did not have a PPM prior to TAVI, the PPM rate was 15.3% (13/85 patients). The majority of patients (12/13) received a PPM due to peri- or post-procedural 3rd degree AV block and one due to 2nd degree type II AV block. After one year 4 more patients had received a new PPM. Three patients due to 3rd degree AV block and one due to an unspecified AV block.

There were 37 patients who developed a LBBB (left bundle branch block) post TAVI. Among the patients who had a PPM implanted after discharge their discharge ECG were as follows; two patients had a 1st degree AV block and LBBB, one had a 1st degree AV block and incomplete LBBB and in one patient the discharge ECG was missing.

## Discussion

Our single-center study demonstrated a high rate of device success with good clinical outcome in a high-risk population with severe symptomatic aortic stenosis receiving the mechanically expandable Lotus Valve System. We also reported low rates of new PPM using a modified implantation technique together with low rates of major stroke, major vascular bleeding and PVL. The ability to fully reposition or retrieve the valve, even in a locked final position meant that we had no cases of valve migration, valve embolization or TAVI-in-TAV deployment.

The need of a PPM after TAVI has not been associated with any increased risk of mortality. [17, 18] It does however add costs of another invasive procedure with associated risk of complications, and delays in patient discharge. High PPM rates after implantation of a Lotus Valve System compared to 1st generation TAVI has been identified as a significant drawback of the technology. [19, 20] The PPM rates in all patients in the REPRISE I and II studies were 36 and 28.6% respectively. [7, 8] Two single-center studies using the Lotus Valve System have demonstrated an incidence of new PPM of 24.1 and 39.9% in pacemaker-naïve patients. [11, 20] A third single-center study presented 28% PPM rate in all patients. [21] Furthermore, the RESPOND post market evaluation study demonstrated 30% new PPM in all patients and 34.6% in pacemaker-naïve patients after implantation of the Lotus Valve System. [14] Krackhardt et al. have previously reported 9.5% PPM in a small case series after using a high implantation technique. [22] We reported 13% PPM rate in all patients due to 2nd degree type II or 3rd degree AV block, and 15.3% in pacemaker-naïve patients. We have demonstrated low PPM rate confirming the benefit of limiting the implantation depth. In the Swedish Percutaneous Valve Registry, where all TAVI in Sweden are registered, the combined reported PPM rate after Lotus Valve implantation in all implanting hospitals in Sweden has been less than 17% since 2013. [23] A possible explanation for the observed low PPM-rates in this study is a careful pre-operative assessment using gated computer tomography to avoid oversizing the Lotus Valve, and the introduction of a new implantation technique where the valve is kept in a high position during the entire deployment aiming to avoid mechanical interaction with the LVOT. Ideal final Lotus Valve position was considered to be with minimal valve protrusion in LVOT while still allowing access of the coronary ostia.

The Lotus Valve System was designed to reduce PVL compared to 1st generation TAVI valves by using an adaptive seal. [19, 20] The occurrence of moderate or severe PVL after TAVI implantation is an independent predictor of increased short and long-term mortality rate. [9, 10, 24, 25] In the REPRISE II CE mark multicenter study where 120 patients received a Lotus Valve System, the rate of PVL at discharge was mild in 13.1% and moderate in 2% of patients. After one year 11.4% of patients had a mild PVL and no patient had a moderate or severe PVL. [8] In a single-center experience including 110 patients there were no patients with moderate/severe PVL, with mild PVL noted in 9.1%. [11] In another single-center experience including 50 Lotus patients moderate/severe PVL was 2% and mild 6%. [21] The corresponding numbers in a newly published single-center experience with 202 Lotus Valves were 0 and 3% respectively. [20] Theoretically, using an implantation technique in which the Lotus Valve interaction with the LVOT is minimized, a higher incidence of PVL could potentially be expected due to less engagement of the adaptive seal in the LVOT. However, we show similar results to other studies with 12% mild PVL and only one case of moderate PVL due to a calcified nodule. This suggests that the modified technique utilized in this patient cohort did not negatively affect PVL.

There are several limitations to this study. First, this was a single-center non-randomized study. Second, TTE measurements were not performed by a central core lab, and follow-up TTE were not available in all patients. The main findings of this small study need to be verified in a larger study.

## Conclusions

In our study the Lotus Valve System demonstrated a high rate of device success and a low rate of procedural mortality, with low rates of procedural complications in a high-risk patient population with severe symptomatic aortic stenosis. By avoiding oversizing the Lotus Valve and introducing a new implantation technique we achieved a low need for new PPM without negatively affecting PVL. These results may provide insight on how to optimize implantation when using the Lotus Valve System.

## Abbreviations

AV: Atrioventricular
ECG: Electrocardiogram
ESC: the european society of cardiology
LAH: Left anterior hemiblock
LBBB: Left bundle branch block
LVEF: Left ventricular ejection fraction
LVOT: Left ventricular outflow tract
PPM: Permanent pacemaker
PVL: Paravalvular leak
RBBB: Right bundle branch block
SAVR: Surgical aortic valve replacement
STS: the society of thoracic surgeons
TAVI: Transcatheter aortic valve implantation
TTE: Transthoracic echocardiogram
VARC: the valve academic research consortium
